# The Science and Art of Medicine

**Published:** 1905-10-21

**Authors:** 


### The Hospital.

A Journal of

The Medical Sciences and Hospital Administration.

Vol. XXXIX.—No. 995. SATURDAY, OCTOBER 21, 1905.

### The Science and Art of Medicine.

The distinction between the pursuit of medicine as a science, and the practice of medicine as an art, a distinction which has been manifest to thinkers since the dawn of history, seems likely, in our own day, to become accentuated by the constantly increasing fascination exercised by the former aspect of what, after all, is in essentials undivisible. We have but lately passed through a period marked by an intense study of symptomatology, that is to say, by a minute observation and careful classification of effects; and we are entering, or, indeed, have actually entered, upon a period which seems likely to be marked by sedulous efforts to refer these effects to their causes, and, in the direction of treatment, to meet them by anticipation rather than to follow them by cure. The trend of modern professional thought, if we may gather that trend from the general character of the introductory addresses recently delivered at the various schools of medicine, is largely in the direction of research after causes; and the time when it was regarded as a legitimate ambition to attach one's name as an observer to a "sign" or a "symptom," is giving way to one in which it will be a chief object of endeavour to discover how the sign or the symptom respectively comes to pass. Research is clearly to be the dominant note in the medical science of the immediate future; and the development of clinical laboratories is regarded as a provision of the machinery from which the greatest results are to be expected. Appeals for the support and for the endowment of research are being made in all directions; and the subscribers to hospitals are being told how infinitely greater and more important are the benefits likely to arise from the laboratories than any which can reasonably be expected from the mere conduct of treatment in the wards. The latter benefits are restricted to the individual patients and to those dependent upon them; the former may well become co-extensive with mankind.

If we glance at the results which have hitherto been obtained in medicine from what may be comprehensively called research, perhaps the first and most striking conclusion which will be arrived at is the apparent want of connection between that research and the art of healing or its professors. Pasteur was, of course, the pioneer of bacteriology in its relations to disease; and Pasteur was a chemist and a biologist, but not a physician. The experiments through which Lord Lister succeeded in rendering the discoveries of Pasteur available for removing the disgrace of excessive mortality from surgery were the experiments of a physicist rather than of a surgeon. The researches which have disclosed the causes and the methods of communication of intermittent and yellow fevers, of elephantiasis, and of sleeping sickness, have been researches in biology and entomology rather than in anything which would ordinarily be described as medicine. The conclusion is irresistible that, as with the sentiment of the Roman poet that nothing incidental to humanity should be alien from the sympathies of man, so nothing in the nature of science, that is to say, of precise and systematised knowledge, should be thought irrelevant to the work of the physician.

If all this be so, it surely follows that the time has come at which some of our time-honoured notions about the character and essentials of medical education should be remodelled by the light of experience, and in which a careful training in general scientific methods should be looked upon as the only safe foundation upon which the complete medical character can be built. The medical student should come to the wards of the hospital, and to the study of clinical medicine and clinical surgery, prepared by some practical knowledge of the meaning of exactness, and of the precautions necessary in order to avoid the vitiation of experiment by accidental error. He requires the training of the physical laboratory to qualify him for the proper conduct of observation at the bedside; and he requires the scientific habit of thought to qualify him for the proper use of hypotheses in relation to the causation of disease. It is of little use to teach him to recognise, for example, what is called "Wernicke's symptom," unless he has been prepared by previous training to use the necessary caution with regard to the direction in which the light is suffered to fall upon the eye; or, in other words, unless he has been educated to the necessity for rigorous exactness in all the details of an experiment. It is of little use to teach him to recognise such conditions as "*tache cérébrale*," or disturbances of the patellar or other reflexes, if he is allowed to regard them as indications of some diseased condition with which they are frequently associated, instead of as phenomena themselves requiring interpretation. It would be difficult, if not impossible, to over-estimate the importance of accuracy and promptitude of clinical investigation; and the time will probably never come when the skill of the clinician as an interpreter will be superseded and rendered unnecessary by mechanical aids to diagnosis. But such accuracy and such promptitude must be matters of secondary rather than of primary acquisition; and it is at least probable, we might almost say certain, that they will be acquired in the greatest fulness by the student whose previous education in physics has been the most thorough and the most complete.

